# Physical activity has a more beneficial effect on the risk of all-cause mortality in patients with metabolic syndrome than in those without

**DOI:** 10.1186/s13098-023-01227-2

**Published:** 2023-12-07

**Authors:** Chang-Hoon Lee, Kyung-Do Han, Min-Sun Kwak

**Affiliations:** 1 Department of Internal Medicine, Seoul National University Hospital, Seoul, Republic of Korea; 2 Department of Statistics and Actuarial Science, Soongsil University, Seoul, Republic of Korea; 3 Department of Internal Medicine, Healthcare Research Institute, Healthcare System Gangnam Center, Seoul National University Hospital, 39FL., Gangnam Finance Center 737, Yeoksam-Dong, Gangnam-Gu, Seoul, 06236 Republic of Korea

**Keywords:** Physical activity, Exercise, Metabolic syndrome, Mortality

## Abstract

**Background:**

It has not been clarified whether physical activity (PA) has more benefit in terms of health outcomes, including mortality risk, among those with metabolic syndrome (MS) compared to those without. Therefore, the aim of this study is to elucidate whether regular PA has interaction with MS on health outcomes.

**Methods:**

Participants with no underlying cardiovascular diseases who underwent national health screening in 2009 were included. According to the metabolic equivalent (MET)-minutes/week, the amount of PA among the participants was grouped as follows: Group 1 (0 MET-minutes/week), Group 2 (1–499), Group 3 (500–999), Group 4 (1000–1499), and Group 5 (≥ 1500). Multivariable Cox proportional hazard models were applied to evaluate the impacts of the amount of PA on health outcomes among those with and without MS. Health outcomes included all-cause mortality and incident cardiovascular diseases (CVDs).

**Results:**

Of 9,628,109 total participants, 335,970 deaths occurred during a median 8.3-year follow-up. After adjustment for age, sex, smoking status, alcohol consumption, and body mass index, the higher the PA amount was, the lower the risk of all-cause mortality in both those with MS [adjusted hazard ratio (aHR) compared with Group 1, 0.86 (95% CI 0.85, 0.87) in Group 2; 0.82 (95% CI 0.81, 0.83) in Group 3; 0.75 (95% CI 0.74, 0.77) in Group 4; and 0.78 (95% CI 0.76, 0.80) in Group 5; *P* for trend < 0.001] and those without MS [aHR compared with Group 1, 0.87 (95% CI 0.86, 0.88) in Group 2; 0.84 (95% CI 0.83, 0.85) in Group 3, 0.79 (95% CI 0.78, 0.80) in Group 4, and 0.82 (95% CI 0.81, 0.84) in Group 5; *P* for trend < 0.001]. The beneficial effects of the amount of PA on all-cause mortality were larger among those with MS than among those without MS in a multiplicative interaction (*P* for interaction < 0.001). The results were similar in the analysis of the relationship between the PA amount and incident CVD.

**Conclusions:**

More PA was associated with a lower risk of all-cause mortality, which was more prominent in those with MS than in those without MS. Physicians should emphasize more the importance of PA in patients with MS.

**Supplementary Information:**

The online version contains supplementary material available at 10.1186/s13098-023-01227-2.

## Introduction

Physical activity (PA) has a beneficial effect on reducing all-cause mortality [1, 2], and current expert guidelines recommend consistent PA of at least 150 min/week of moderate-intensity or 75 min/week of vigorous-intensity aerobic PA for adults [3]. However, roughly half of the real world population does not meet the recommended amount of PA [4]. In particular, those with cardiovascular diseases (CVDs) were reported to be more sedentary and less active than healthy individuals [5, 6], possibly because of physical unfitness, even though individuals with CVD may benefit from PA to a greater extent than healthy subjects without CVD [7]. Therefore, studies that investigate whether earlier encouragement of PA could be helpful to those at risk of CVD are needed. We previously reported that sustained PA was associated with a decreased risk of all-cause mortality in those with hypertension or diabetes mellitus, who are at a high risk of CVD, more strongly than in those without hypertension or diabetes mellitus. [8] However, whether PA is more helpful for those with metabolic syndrome (MS) than for those without MS has not yet been clarified.

MS is a cluster of cardio-metabolic risk factors including abdominal obesity, insulin resistance, hypertension, and hyperlipidemia. The estimated global prevalence of MS is approximately one-quarter of the world population, affecting more than a billion people worldwide [9]. Considering that PA is beneficial to those with cardiovascular diseases and MS is strongly linked to cardiovascular diseases [10], it is crucial to determine whether PA is also beneficial and is even more helpful to those with MS. Therefore, the aim of this study is to elucidate whether regular PA has interactions with MS on health outcomes, including the risk of all-cause mortality.

## Methods

### Data source: the national health insurance service database

We used the National Health Insurance Service (NHIS) database, which covers nearly all Koreans (97.2% of the Korean population) and is managed by the Korean government. The NHIS encourages annual or biennial standardized health check-ups for all insured Koreans older than 40 years and employees older than 20 years. The NHIS contains the following information for each participant: demographics; lifestyle behaviors, including a PA questionnaire; anthropometric measurements; examinations; medical history, such as claims for disease diagnosis codes of the International Classification of Diseases (ICD-10); and treatments, including procedures performed and medications prescribed.

This study protocol was conducted in accordance with the ethics guidelines of the Helsinki Declaration of 1975 and was given an exemption by the Institutional Review Board of Seoul National University Hospital (H-2211-004-1373). The requirement for informed consent was waived because of the retrospective study design, and the researchers only accessed anonymous data for analytical purposes.

### Study population

**Fig. 1:**
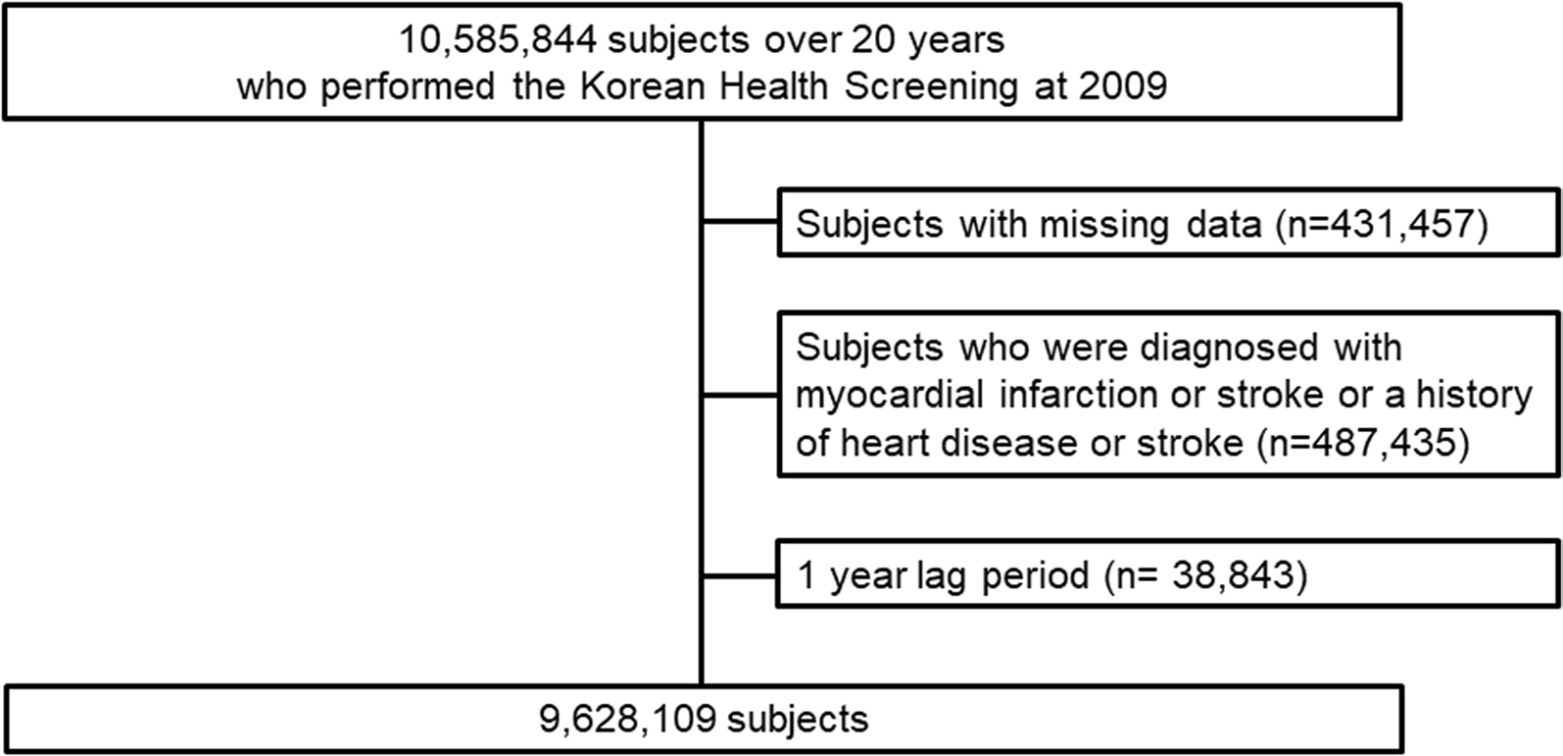
Flowchart of the study enrollment

Participants older than 20 years of age who underwent the Korean Health Screening in 2009 were initially included. Among them, participants with missing data were excluded. In addition, participants who were diagnosed with a major cardiovascular disease (stroke (I63, I64) or myocardial infarction (I21, I22)) or who had a history of major cardiovascular disease (heart disease or stroke) based on a questionnaire were excluded. Then, participants who died or had an event within 1 year (1 lag period) were also excluded. The included participants were followed up until December 2018 (Fig. 1).

### PA measurements

The PA questionnaire used by the Korean National Health Insurance Service (KNHIS) in 2009 was the last 7-day recall and is a well-validated questionnaire for national monitoring [11, 12]. Each question is used to determine the frequency of light, moderate and vigorous PA. Each individual marked how many days during the last 7 days they performed each grade of PA (Additional file 1: Table S1) [12]. To estimate the overall amount of PA in a quantitative manner, we calculated the metabolic equivalent (MET), which is widely used in medical research to quantitatively measure PA amount [13, 14]. According to The 2011 Updates on Compendium of PA, we appropriated 8, 5 and 3 METs to each question, and MET-minutes per week were calculated as follows: MET value x minutes spent per week [12–14]. According to MET-minutes/week, PA amount was grouped as follows: Group 1 (0), Group 2 (1-499), Group 3 (500-999), Group 4 (1000-1499), and Group 5 (≥ 1500 MET-minutes/week).

### Diagnosis of MS

The diagnosis of MS was defined according to the harmonizing criteria [15]. MS was diagnosed if 3 or more of the following components were present: (1) increased waist circumference (≥ 90 for Asian men and ≥ 80 cm for Asian women); (2) high blood triglycerides (≥ 150 mg/dL) or use of a relevant drug; (3) low high-density lipoprotein cholesterol level (< 40 mg/dL for men and 50 mg/dL for women) or use of a relevant drug; (4) high blood pressure (systolic ≥ 130 mmHg and/or diastolic ≥ 80 mmHg) or use of an antihypertensive medication; and (5) high blood sugar level (≥ 100 mg/dL) or use of an antidiabetic drug.

### Measurement of clinical parameters and biochemical analysis

The standardized self-administered questionnaires included age, sex, smoking status, alcohol consumption, annual income, PA and underlying diseases.

Height (m) and body weight (kg) were measured using an electronic scale. BMI was calculated as follows: BMI = body weight (kg)/height^2^ (m^2^). The waist circumference (WC) was measured using a tape measure at the midpoint between the lower costal margin and the iliac crest by a trained examiner. The systolic blood pressure (SBP) and diastolic blood pressure (DBP) were measured after 5 min of rest.

Blood samples were collected after overnight fasting. The biochemical analysis included assessments of serum fasting glucose, total cholesterol, high-density lipoprotein cholesterol, low-density lipoprotein cholesterol and triglycerides. All laboratory analyses were performed using standardized laboratory methods.

The diagnoses of hypertension, diabetes mellitus (DM) and dyslipidemia were defined using anthropometric measurements and laboratory data (SBP 140 mmHg or DBP 90 mmHg; fasting glucose level ≥ 126 mg/dL; total cholesterol levels ≥ 240 mg/dL) or ICD codes (ICD I10 to I13 or I15; E11 to E14; E78) and medication use, including antihypertensive medication, insulin or oral hypoglycemic agents, and dyslipidemia medication.

### Outcomes

The primary outcome was all-cause mortality. The NHIS database includes whether a person died during the follow-up duration of the study (until 31 Dec 2018) and the death date if a person died. The secondary outcome was incident CVD, including myocardial infarction and stroke. Stroke was defined when the ICD code I63 or I64 was used with hospitalization and when a claim for an imaging study, including magnetic resonance imaging or computed tomography, was made. Myocardial infarction was diagnosed when ICD code I21 or I22 was used with hospitalization.

### Statistical analysis

Continuous variables are expressed as the means ± standard deviations, and categorical variables are expressed as numbers and percentages. Between-group comparisons were performed using Student’s t test or a one-way analysis of variance for continuous variables and chi-square tests for categorical variables. Log transformation was performed for nonnormally distributed variables. All-cause mortality or incident cardiovascular disease was calculated as the number of events per 1,000 total person-years. Multivariable Cox proportional hazards regression analyses were performed after adjustment for covariates, in which estimates were presented as adjusted hazard ratios (aHRs) with 95% confidence intervals (CIs).

The interaction term “presence of MS” x “amount of PA” was analyzed to evaluate whether there was a multiplicative interaction between the presence of MS and the amount of PA on all-cause mortality.

Statistical analyses were performed using R version 3.2.3 (The R Foundation for Statistical Computing, Vienna, Austria) and SAS version 9.4 (SAS Institute Inc., Cary, NC, USA). A two-sided *P* value less than 0.05 was considered statistically significant.

## Results

In total, 9,628,109 participants (mean age, 46.3 ± 13.7 years; 55.3% were male) were included in the analysis. Figure 1 shows the flowchart of the study enrollment. The baseline characteristics of the total population and participants according to the amount of PA are presented in Table 1. Additional file 1: Table S2 shows the baseline characteristics of the participants according to the presence of MS.

**Table 1:** Baseline characteristics according to the amount of physical activity (MET-minutes/week)

	Total	Physical activity (MET-minutes/week)					
		Totally sedentary	1–499	500–999	1000–1499	≥ 1500	*P*-value
N	9628109	2301377	3165137	2675670	997256	488669	
Age, years	46.26 ± 13.73	48.83 ± 14.16	44.33 ± 13.23	45.42 ± 13.67	46.86 ± 13.18	50 ± 13.61	< 0.001
Men	5319688 (55.3)	1123340 (48.8)	1712303 (54.1)	1566254 (58.5)	611380 (61.3)	306411 (62.7)	< 0.001
Current smoker	2579462 (26.7)	564096 (24.5)	864992 (27.3)	765,867 (28.6)	262603 (26.3)	121904 (25.0)	< 0.001
Alcohol consumption	4767220 (49.5)	865478 (37.6)	1636801 (51.7)	1467412 (54.8)	546423 (54.8)	251106 (51.4)	< 0.001
Lowest quartile of income (Q1)	1686829 (17.5)	439357 (19.1)	518625 (16.4)	461743 (17.3)	177049 (17.8)	90055 (18.4)	< 0.001
BMI (kg/m^2^)	23.7 ± 3.5	23.6 ± 3.3	23.5 ± 3.3	23.7 ± 3.2	24.0 ± 4.9	24.1 ± 3.0	< 0.001
BMI ≥ 25 kg/m^2^	3110732 (32.3)	738993 (32.1)	985471 (31.1)	867244 (32.4)	345125 (34.6)	173899 (35.6)	< 0.001
WC (cm)	80.2 ± 9.4	80.1 ± 9.5	79.7 ± 9.8	80.2 ± 9.3	80.7 ± 9.0	81.1 ± 8.6	< 0.001
SBP (mmHg)	122.2 ± 14.9	122.83 ± 15.54	121.24 ± 14.67	122.14 ± 14.73	122.8 ± 14.55	123.81 ± 14.83	< 0.001
DBP (mmHg)	76.2 ± 10.0	76.45 ± 10.22	75.83 ± 9.99	76.25 ± 9.98	76.59 ± 9.84	76.91 ± 9.88	< 0.001
Hypertension	2289895 (23.8)	613844 (26.7)	660946 (20.9)	619812 (23.2)	251558 (25.2)	143735 (29.4)	
Dyslipidemia	1628438 (16.9)	416946 (18.1)	503714 (15.9)	439560 (16.4)	175615 (17.6)	92603 (19.0)	< 0.001
Diabetes mellitus	766064 (8.0)	199757 (8.7)	216009 (6.8)	208234 (7.8)	87335 (8.8)	54729 (11.2)	< 0.001
Fasting glucose (mg/dL)	96.8 ± 23.3	97.4 ± 24.6	96 ± 22.5	96.7 ± 22.9	97.4 ± 23.1	98.8 ± 24.5	< 0.001
TC (mg/dL)	195.3 ± 41.2	196.4 ± 42.7	194.9 ± 41.1	194.8 ± 40.2	195.5 ± 40.4	195.7 ± 40.6	< 0.001
LDL (mg/dL)	113.6 ± 38.7	114.5 ± 41.3	113.1 ± 37.7	113.1 ± 37.9	113.8 ± 38.0	114.1 ± 38.4	< 0.001
HDL (mg/dL)	56.6 ± 32.7	57.8 ± 42.9	56.0 ± 28	56.3 ± 29.2	56.4 ± 28.2	56.8 ± 31.7	< 0.001
Triglyceride (mg/dL)*	112.07 (112.03–112.11)	114.53 (114.45–114.62)	112.1 (112.03–112.17)	111.11 (111.04–111.19)	110.61 (110.48–110.73)	108.78 (108.61–108.96)	< 0.001
Metabolic syndrome	2253690 (23.4)	591380 (25.7)	691591 (21.9)	607456 (22.7)	237353 (23.8)	125910 (25.8)	< 0.001

Categorical variables are expressed as number (%); continuous variables are expressed mean ± standard deviation

*BMI* body mass index, *WC* waist circumference, *SBP* systolic blood pressure, *DBP* diastolic blood pressure, *TC* total cholesterol, *LDL* low-density lipoprotein, *HDL* high-density lipoprotein, *MET* metabolic equivalent

^*^Geometric means (95% confidence interval)

The median follow-up duration of this cohort was 8.3 years (interquartile range, 8.1- 8.6). A total of 335,970 (4.23 incidence rate per 1000 person-years) participants died during follow-up. Table 2 and Additional file 1: Figure S1 show that greater amounts of PA were associated with lower all-cause mortality regardless of the presence of MS after adjustment for age, sex, smoking status, alcohol consumption, and body mass index (aHR, 0.87; 95% CI, 0.86, 0.88 for Group 2, aHR, 0.84; 95% CI 0.83, 0.85 for Group 3, aHR 0.79, 95% CI 0.78, 0.80 for Group 4, and aHR 0.82, 95% CI 0.81, 0.84 for Group 5 compared to Group 1 in participants without MS, *P* for trend < 0.001; aHR, 0.86; 95% CI 0.85, 0.87 for Group 2, aHR 0.82, 95% CI 0.81, 0.83 for Group 3, aHR 0.75; 95% CI 0.74, 0.77 for Group 4, and 0.78, 95% CI 0.76, 0.80 for Group 5 in participants with MS, *P* for trend < 0.001). When the interaction of MS on the relationship of PA and mortality was evaluated, there was a significant multiplicative interaction (*P* for interaction < 0.001).

**Table 2:** The effect of amount of physical activity on all-cause mortality and incidence of cardiovascular disease according to the presence of metabolic syndrome

Presence of metabolic syndrome	MET score	N	Events	Person-years	Incidence rate per 1000	Univariate model	Multivariate model 1	Multivariate model 2	Multivariate model 3
All-cause mortality									
MS (−)	Totally sedentary	1709997	67818	14057952.64	4.824	1 (reference)	1 (reference)	1 (reference)	1 (reference)
	1–499	2473546	53916	20456279.16	2.636	0.55 (0.54, 0.55)	0.85 (0.84, 0.86)	0.87 (0.86, 0.88)	0.87 (0.86, 0.88)
	500–999	2068214	51171	17110004.09	2.990	0.62 (0.61, 0.63)	0.81 (0.80, 0.82)	0.84 (0.83, 0.85)	0.84 (0.83, 0.85)
	1000–1499	759903	18762	6297310.76	2.979	0.62 (0.61, 0.63)	0.74 (0.73, 0.75)	0.79 (0.78, 0.80)	0.79 (0.78, 0.80)
	≥ 1500	362759	13157	2996779.46	4.390	0.91 (0.89, 0.93)	0.76 (0.75, 0.78)	0.82 (0.81, 0.84)	0.83 (0.81, 0.84)
*P* for trend						< 0.001	< 0.001	< 0.001	< 0.001
MS ( +)	Totally sedentary	591380	45942	4809944.99	9.551	1 (reference)	1 (reference)	1 (reference)	1 (reference)
	1–499	691591	33941	5672370.44	5.984	0.63 (0.62, 0.64)	0.85 (0.83, 0.86)	0.86 (0.85, 0.87)	0.86 (0.85, 0.87)
	500–999	607456	31689	4981576.91	6.361	0.67 (0.66, 0.68)	0.80 (0.79, 0.81)	0.82 (0.81, 0.83)	0.82 (0.81, 0.83)
	1000–1499	237353	11466	1951561.18	5.875	0.62 (0.60, 0.63)	0.72 (0.71, 0.74)	0.75 (0.74, 0.77)	0.76 (0.74, 0.77)
	≥ 1500	125910	8108	1030530.11	7.868	0.82 (0.81, 0.84)	0.74 (0.72, 0.76)	0.78 (0.76, 0.80)	0.78 (0.77, 0.80)
*P* for trend						< 0.001	< 0.001	< 0.001	< 0.001
*P* for interaction						< 0.001	0.248	< 0.001	< 0.001
Incident cardiovascular diseases									
MS (−)	Totally sedentary	1709997	33603	13950759.84	2.409	1 (reference)	1 (reference)	1 (reference)	1 (reference)
	1–499	2473546	29232	20362317.67	1.436	0.60 (0.59, 0.61)	0.84 (0.83, 0.86)	0.87 (0.85, 0.88)	0.89 (0.87, 0.90)
	500–999	2068214	27174	17022721.37	1.596	0.66 (0.65, 0.67)	0.82 (0.80, 0.83)	0.84 (0.83, 0.86)	0.85 (0.81, 0.87)
	1000–1499	759903	10386	6263598.83	1.658	0.69 (0.67, 0.70)	0.78 (0.76, 0.79)	0.82 (0.80, 0.83)	0.83 (0.81, 0.84)
	≥ 1500	362759	6790	2974979.44	2.282	0.95 (0.92, 0.97)	0.81 (0.79, 0.83)	0.85 (0.83, 0.87)	0.85 (0.83, 0.87)
*P* for trend						< 0.001	< 0.001	< 0.001	< 0.001
MS ( +)	Totally sedentary	591380	29762	4712057.42	6.316	1 (reference)	1 (reference)	1 (reference)	1 (reference)
	1–499	691591	24811	5590106.32	4.438	0.70 (0.69, 0.71)	0.90 (0.88, 0.92)	0.91 (0.90, 0.93)	0.89 (0.88, 0.91)
	500–999	607456	22478	4907373.12	4.580	0.73 (0.71, 0.74)	0.84 (0.83, 0.86)	0.87 (0.85, 0.88)	0.86 (0.84, 0.87)
	1000–1499	237353	8280	1923443.14	4.305	0.68 (0.66, 0.70)	0.77 (0.75, 0.79)	0.81 (0.79, 0.83)	0.80 (0.78, 0.82)
	≥ 1500	125910	5384	1012460.19	5.318	0.84 (0.82, 0.87)	0.77 (0.75, 0.79)	0.81 (0.79, 0.84)	0.82 (0.80, 0.84)
*P* for trend						< 0.001	< 0.001	< 0.001	< 0.001
*P* for interaction						< 0.001	< 0.001	< 0.001	< 0.001

Multivariate model 1 was adjusted for age and sex

Multivariate model 2 was adjusted for age, sex, smoking, alcohol consumption, and body mass index

Multivariate model 3 was adjusted for age, sex, smoking, alcohol consumption, income and body mass index

*MET* metabolic equivalent, *MS* metabolic syndrome, *CI* confidence interval

This trend was the same for incident CVD. As the amount of PA increased, incident CVD decreased regardless of the presence of MS (*P* for trend < 0.001 for both with and without MS groups). There was also a significant multiplicative interaction (*P* for interaction < 0.001) of the presence of MS on the relationship between PA and incident CVD.

**Fig. 2:**
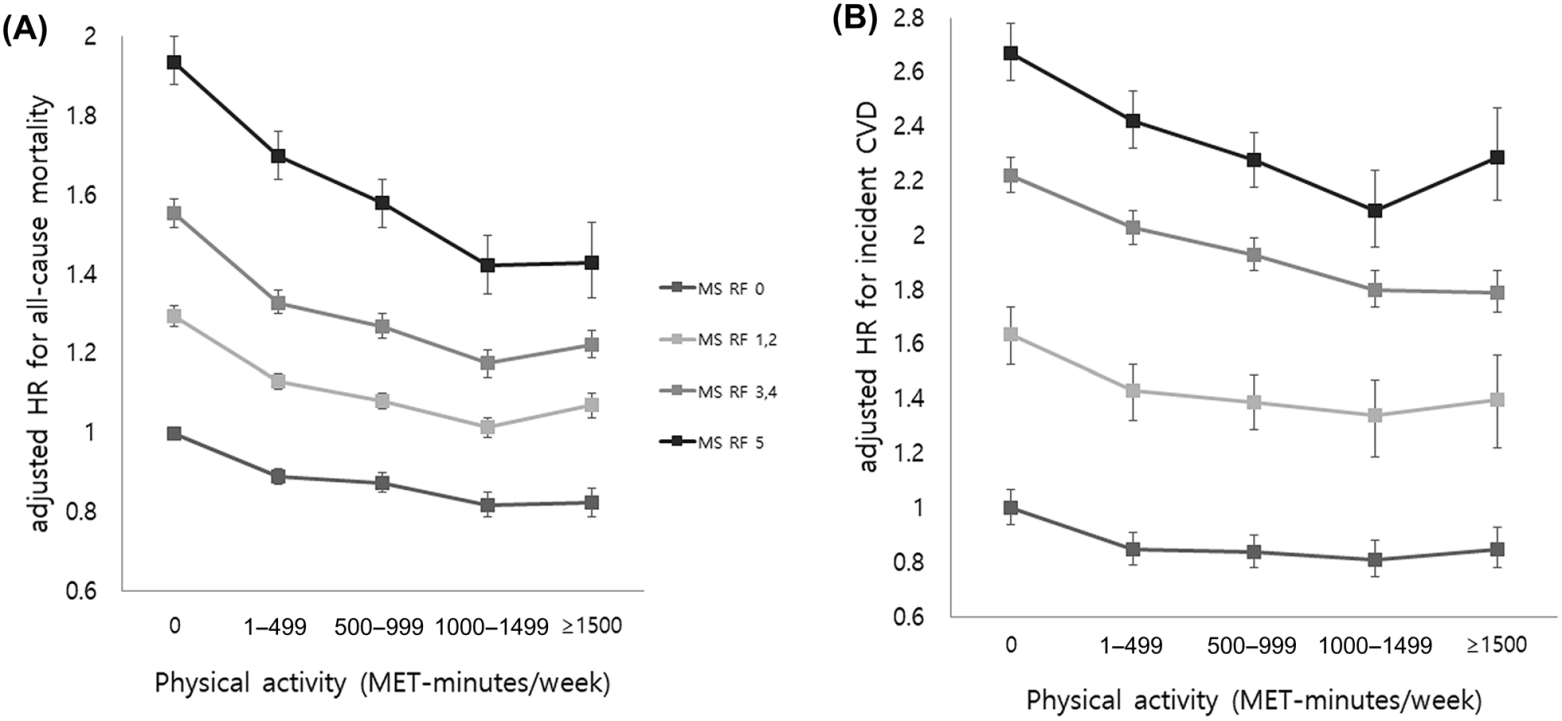
The multivariable-adjusted risk for **a** all-cause mortality and **b** incident cardiovascular disease by physical activity amount according to the number of metabolic syndrome components after adjustment for age, sex, smoking, drinking, and body mass index. *HR* hazard ratio, *CVD* cardiovascular disease, *MS* metabolic syndrome, *METs* metabolic equivalent, *RF* risk factor

A similar trend was noted in that all-cause mortality and incident CVD decreased as the amount of PA increased, and the inclination was steeper in the participants with more risk factors for MS (Fig. 2 and Additional file 1: Tables S3 and S4).

## Discussion

This large population-based cohort study shows that those with MS get more benefit in reduced all-cause mortality risk from PA than those without MS. PA was also associated with a decreased risk of incident CVD in individuals with MS to a greater extent than in those without MS.

It has been reported that those with MS have a higher risk of CVD outcomes and all-cause mortality compared to those without MS [16, 17]. This finding suggests that prevention and management of MS are important. The current mainstay of treatment remains lifestyle modification with PA and diet [18]. PA reforms body composition, reduces body mass index and waist circumference, and improves metabolic and cardiovascular profiles including fasting blood glucose or blood pressure in people with MS [19]. Moderate-to-vigorous PA is also associated with a reduced risk of major cardiovascular events and mortality in those with MS [16]. However, it has not yet been studied whether PA is more effective in those with MS compared to those without MS in terms of reducing all-cause mortality risk. Consistent with previous studies, this study showed that PA is associated with reduced all-cause mortality in a dose-dependent manner both in those with and without MS (Table 2). Those with MS who engaged in 1–499 MET-minutes/week had a 14% lower risk of mortality, while those who performed 500–999 MET-minutes/week had an 18% lower risk, and those who engaged in 1000–1499 MET-minutes/week had a 24% lower risk of all-cause mortality. In those without MS, the reduced risks of all-cause mortality for the three PA groups were 13%, 16% and 21%, respectively. The analysis revealed that the presence of MS has a multiplicative interaction with PA on the effects of decreased risk of all-cause mortality. To our knowledge, this is the first study demonstrating that those with MS can obtain even greater benefits in reduced all-cause mortality risk from PA than those without MS. Additionally, our study also shows that PA is more helpful to those with MS compared with those without MS for a decreased risk of CVD incidence. The majority of the observed mortality reduction in those with MS who performed sufficient PA could have resulted from a reduced CVD risk. The results of this study demonstrate that those with MS, a more common and larger group of individuals with pre-CVD status, should be encouraged to engage in PA.

The biologic mechanism for how PA has a more beneficial effect on longevity in patients with MS than in those without MS has not been clarified. Several plausible explanations are as follows. First, PA can improve the metabolic profile, including insulin sensitivity, hepatic glucose production or utilization of fatty acids, which is worse in patients with MS compared to those without MS [8]. Thus, the effect of PA might be more dramatic in individuals with MS. Second, sarcopenia is closely associated with MS and physical inactivity [20] as insulin resistance in MS is associated with hyperinsulinemia and elevated myostatin levels, both of which reduce skeletal muscle mass [21]. PA has a positive effect on muscle mass and muscle function [22, 23], and skeletal muscle is a potential antioxidant organ during inflammatory reactions such as those associated with MS. This positive effect of PA on sarcopenia might contribute to increased longevity, especially in patients with MS. Third, PA can reduce systemic inflammation and oxidative stress, which can lead to endothelial dysfunction, arterial stiffness, and vascular dysfunction; thus, PA can reduce the risk of micro- and macrovascular complications [8, 24]. As patients with MS are more vulnerable to cardiovascular disease directly because of inflammatory pathways or indirectly via increased production of reactive oxygen species, endothelial dysfunction, and vascular dysfunction [25], PA in these individuals could contribute to the improving of these phenomena, thus extending longevity.

In this study, more than half of the study participants did not reach the recommended level of PA. Intriguingly, the effect of decreasing mortality risk was also observed among those who performed PA at the level of less than recommended in this study. The mortality risk decreased most steeply in the group with the lowest level of PA (1–499 MET-minutes/week PA) (Table 2 and Additional file 1: Figure S1). This trend was similar in both with or without MS. In fact, previous studies also reported that a lesser amount of PA than what is currently recommended can still have significant health benefits [26]. A study showed that running, even with a slow speed and for a short duration, was associated with markedly reduced risks of all-cause mortality and cardiovascular disease [27]. In our study, the proportion of individuals who were completely sedentary was higher in the group with MS than in the group without MS (26.2% vs. 23.2%). These findings suggest that even a small amount of PA can lead to a significant health benefit, which can provide good motivation for the people who need PA, including those with MS.

There have not been consistent results regarding the upper limit of PA for obtaining health benefits. Whether the dose response relationship between PA levels and health benefits is curvelinear [28], U-shaped [29, 30], or reverse-J shaped [1] is still under debate. One study suggested that the optimal amount of PA is 960–2400 MET-minutes/week and that no additional benefit can be obtained above this level of PA [26]. Another study showed that strenuous runners have statistically similar mortality rates as sedentary nonrunners and reduced longevity compared to mild or moderate runners [29]. The present study showed a plateau or slight increase of the mortality in the group with ≥ 1500 MET-minutes/week compared to the other groups with lower amounts of PA, although the group with the largest amount of PA obtained more benefits than the sedentary group. There was no difference in this trend according to the presence of MS. This may suggest that extreme PA may be associated with a partial loss of health benefits. However, it should be interpreted more cautiously with additional objectively collected data such as accelerometers. Also, more detailed segmentation of higher levels of PA and additional epidemiological and interventional studies may be warranted to confirm whether the dose–response relationship between PA levels and health benefits is curvilinear, U-shaped, or inverted J-shaped.

The strengths of this study are as follows. First, this study is a large-scale population-based study with strong statistical power to show the interaction between MS and PA on longevity. Second, this is the first study focusing on the interaction of PA and MS on all-cause mortality. This study result might be helpful to emphasize and encourage PA among individuals with MS. We also admit that this study had limitations. First, the amount of PA was measured based on the self-declared 7-day recall questionnaire, which might be affected by recall bias. However, the 7-day recall survey has been widely used and is well validated in many previous studies. Second, cause-specific death was not evaluated in this study. However, judging from the similar trend in the incident CVD results, mortality from CVD seems to have had a great influence on all-cause mortality. Third, this study does not provide a specific type of exercise that is most effective in reducing negative outcomes in people with MS. Further studies should be conducted.

In conclusion, those with MS may experience even greater longevity-extending benefits from PA than those without MS. Physicians should place greater emphasis on the importance of PA, especially in those with MS, and encourage them to engage in PA.

### Supplementary Information

**Additional file 1: Table S1.** Questionnaires on the habits of leisure-time physical activity evaluated during routine health check-ups by National Health Insurance Service. **Table S2.** Baseline characteristics according to the presence of metabolic syndrome. **Table S3.** The effect of the number of metabolic syndrome components and physical activity amount on all-cause mortality. **Table S4.** The effect of the number of metabolic syndrome components and PA amount on cardiovascular disease. **Figure S1.** The multivariable-adjusted risk for (a) all-cause mortality and (b) incident cardiovascular disease by physical activity amount according to the presence of metabolic syndrome after adjustment for age, sex, smoking status, alcohol consumption, and body mass index.

## Data Availability

The datasets used and/or analysed during the current study available from the corresponding author on reasonable request.
