# Supplementary material for: Physical activity has a more beneficial effect on the risk of all-cause mortality in patients with metabolic syndrome than in those without
Source: Diabetol Metab Syndr. 2023 Dec 7;15:255. doi: 10.1186/s13098-023-01227-2 (PMC10702028; doi:10.1186/s13098-023-01227-2)

**Additional Files**

**Additional Table 1.** Questionnaires on the habits of leisure-time physical activity evaluated during routine health check-ups by National Health Insurance Service.

| For each question, please check one of the items corresponding to your status of physical activity during the past week | | |
| --- | --- | --- |
| Questions | Examples | Items (number of days) |
| During the recent week,  Question 1. How many days during the past week did you do vigorous physical activities, making you much more breathless than usual, for at least 20 minutes? | Running, aerobics, climbing, biking at a fast pace, mountain climbing | □0 □1 □2 □3 □4 □5 □6 □7 |
| Question 2. How many days during the past week did you do moderate-intensity physical activities making you a little more breathless than usual, for at least 30 minutes?  *Excluding activities included in Question 1. | Brisk walking, tennis doubles, biking at a regular pace | □0 □1 □2 □3 □4 □5 □6 □7 |
| Question 3. How many days during the past week did you walk for a total of 30 minutes or more (when adding up cases with more than 10 minutes of walking)?  *Excluding activities included in Question1 and Question 2. | Light activity, usual walking during commute | □0 □1 □2 □3 □4 □5 □6 □7 |

**Additional Table 2.** Baseline characteristics according to the presence of metabolic syndrome.

|  | Total population | No metabolic syndrome | Metabolic syndrome | *P*-value |
| --- | --- | --- | --- | --- |
| N | 9628109 | 7374419 | 2253690 |  |
| Age, years | 46.26 ± 13.73 | 44.1 ± 13.2 | 53.4 ± 12.9 | <0.001 |
| Men | 5319688 (55.3) | 3979362 (54.0) | 1340326 (59.5) | <0.001 |
| Current Smoker | 2579462 (26.79) | 1960559 (26.6) | 618903 (27.5) | <0.001 |
| Alcohol consumption | 4767220 (49.5) | 3705622 (50.3) | 1061598 (47.1) | <0.001 |
| Lowest quartile of income (Q1) | 1686829 (17.5) | 1303456 (17.7) | 383373 (17.0) | <0.001 |
| BMI (kg/m^2^) | 23.7 ± 3.5 | 23.0 ± 3.0 | 26.0 ± 3.8 | <0.001 |
| BMI ≥ 25kg/m^2^ | 3110732 (32.3) | 1727332 (23.4) | 1383400 (61.4) | <0.001 |
| Waist circumference (cm) | 80.2 ± 9.4 | 77.9 ± 8.5 | 87.3 ± 8.9 | <0.001 |
| SBP (mmHg) | 122.2 ± 14.9 | 119.2 ± 13.8 | 131.7 ± 14.6 | <0.001 |
| DBP (mmHg) | 76.2 ± 10.0 | 74.6 ± 9.4 | 81.5 ± 10.0 | <0.001 |
| Hypertension | 2289895 (23.8) | 1021962 (13.9) | 1267933 (56.3) |  |
| Dyslipidemia | 1628438 (16.9) | 690190 (9.4) | 938248 (41.6) | <0.001 |
| Diabetes mellitus | 766064 (8.0) | 230485 (3.1) | 535579 (23.8) | <0.001 |
| Fasting glucose (mg/dL) | 96.8 ± 23.3 | 92.4 ± 17.0 | 111.2 ± 33.3 | <0.001 |
| Total cholesterol (mg/dL) | 195.3 ± 41.2 | 192.0 ± 38.0 | 206.2 ± 48.6 | <0.001 |
| LDL (mg/dL) | 113.6 ± 38.7 | 122.1 ± 241.7 | 119.7 ± 123.7 | <0.001 |
| HDL (mg/dL) | 56.6 ± 32.7 | 58.6 ± 32.8 | 50.0 ± 31.6 | <0.001 |
| Triglyceride (mg/dL)* | 112.07 (112.03, 112.11) | 97.2 (97.16, 97.24) | 178.57 (178.45, 178.7) | <0.001 |
| Physical activity (MET-minutes/week) | |  |  | <0.001 |
| Totally sedentary | 2301377 (23.9) | 1709997 (23.2) | 591380 (26.2) |  |
| 1-499 | 3165137 (32.9) | 2473546 (33.5) | 691591 (30.7) |  |
| 500-999 | 2675670 (27.8) | 2068214 (28.1) | 607456 (27.0) |  |
| 1000-1499 | 997256 (10.4) | 759903 (10.3) | 237353 (10.5) |  |
| ≥1500 | 997256 (10.4) | 759903 (10.3) | 237353 (10.5) |  |

*Geometric means (95% confidence interval)

Categorical variables are expressed as number (%); continuous variables are expressed mean ± standard deviation

BMI, body mass index; SBP, systolic blood pressure; DBP, diastolic blood pressure; LDL, low-density lipoprotein; HDL, high-density lipoprotein; MET, metabolic equivalent

**Additional Table 3.** The effect of the number of metabolic syndrome components and physical activity amount on all-cause mortality.

| Number of metabolic syndrome components | MET Score | N | Events | Person-years | Incidence rate per 1000 | Univariate Model | Multivariate model 1 | Multivariate model 2 |
| --- | --- | --- | --- | --- | --- | --- | --- | --- |
| 0 | Totally sedentary | 587547 | 11737 | 4862307.48 | 2.414 | 1 (reference) | 1 (reference) | 1 (reference) |
|  | 1-499 | 956309 | 10868 | 7938377.46 | 1.369 | 0.57 (0.55, 0.58) | 0.87 (0.85, 0.89) | 0.89 (0.87, 0.91) |
|  | 500-999 | 773347 | 10192 | 6424692.88 | 1.586 | 0.66 (0.64, 0.68) | 0.84 (0.82, 0.87) | 0.88 (0.85, 0.90) |
|  | 1000-1499 | 268990 | 3674 | 2239273.49 | 1.641 | 0.68 (0.65, 0.70) | 0.76 (0.73, 0.79) | 0.82 (0.79, 0.85) |
|  | ≥1500 | 120551 | 2317 | 1002806.76 | 2.311 | 0.95 (0.91, 1.00) | 0.76 (0.73, 0.80) | 0.83 (0.79, 0.86) |
| *P* for trend |  |  |  |  |  | <0.001 | <0.001 | <0.001 |
| 1,2 | Totally sedentary | 1122450 | 56081 | 9195645.15 | 6.099 | 2.52(2.47, 2.57) | 1.17 (1.14, 1.19) | 1.29 (1.27, 1.32) |
|  | 1-499 | 1517237 | 43048 | 12517901.71 | 3.439 | 1.43(1.40, 1.45) | 0.99 (0.97, 1.01) | 1.13 (1.11, 1.15) |
|  | 500-999 | 1294867 | 40979 | 10685311.21 | 3.835 | 1.59 (1.56, 1.62) | 0.94 (0.92, 0.96) | 1.08 (1.06, 1.10) |
|  | 1000-1499 | 490913 | 15088 | 4058037.27 | 3.718 | 1.54 (1.50, 1.58) | 0.86 (0.84, 0.88) | 1.02 (0.99, 1.04) |
|  | ≥1500 | 242208 | 10840 | 1993972.7 | 5.436 | 2.25 (2.19, 2.31) | 0.89 (0.87, 0.91) | 1.07 (1.04, 1.10) |
| *P* for trend |  |  |  |  |  | <0.001 | <0.001 | <0.001 |
| 3,4 | Totally sedentary | 527190 | 39672 | 4290254.15 | 9.247 | 3.81 (3.74, 3.89) | 1.22 (1.19, 1.24) | 1.56 (1.52, 1.59) |
|  | 1-499 | 624978 | 29305 | 5128930.69 | 5.714 | 2.36 (2.31, 2.42) | 1.03 (1.00, 1.05) | 1.33 (1.3, 1.36) |
|  | 500-999 | 548555 | 27579 | 4500766.62 | 6.128 | 2.54 (2.48, 2.59) | 0.97 (0.95, 0.99) | 1.27 (1.24, 1.30) |
|  | 1000-1499 | 214393 | 10057 | 1763506.96 | 5.703 | 2.36 (2.30, 2.42) | 0.88 (0.86,0.91) | 1.18 (1.14, 1.21) |
|  | ≥1500 | 112794 | 7102 | 923374.43 | 7.691 | 3.18 (3.08, 3.27) | 0.91 (0.88,0.94) | 1.22 (1.19, 1.26) |
| *P* for trend |  |  |  |  |  | <0.001 | <0.001 | <0.001 |
| 5 | Totally sedentary | 64190 | 6270 | 519690.83 | 12.065 | 4.97 (4.82, 5.12) | 1.29 (1.25,1.33) | 1.94 (1.88, 2.00) |
|  | 1-499 | 66613 | 4636 | 543439.75 | 8.531 | 3.52 (3.41, 3.65) | 1.12 (1.08,1.16) | 1.70 (1.64, 1.76) |
|  | 500-999 | 58901 | 4110 | 480810.29 | 8.548 | 3.53 (3.41, 3.66) | 1.03 (1.00,1.07) | 1.58 (1.52, 1.64) |
|  | 1000-1499 | 22960 | 1409 | 188054.22 | 7.493 | 3.10 (2.93, 3.27) | 0.91 (0.86,0.97) | 1.42 (1.35, 1.50) |
|  | ≥1500 | 13116 | 1006 | 107155.67 | 9.388 | 3.87 (3.63, 4.13) | 0.92 (0.86,0.98) | 1.43 (1.34, 1.53) |
| *P* for trend |  |  |  |  |  | <0.001 | <0.001 | <0.001 |

MET, metabolic equivalent

Multivariate model 1 was adjusted for age and sex.

Multivariate model 2 was adjusted for age, sex, smoking, alcohol consumption, and body mass index

**Additional Table 4.** The effect of the number of metabolic syndrome components and PA amount on cardiovascular disease.

| Number of metabolic syndrome components | MET Score | N | Events | Person-years | Incidence rate per 1000 | Univariate Model | Multivariate model 1 | Multivariate model 2 |
| --- | --- | --- | --- | --- | --- | --- | --- | --- |
| 0 | Totally sedentary | 587547 | 4992 | 4846577.15 | 1.030 | 1 (reference) | 1 (reference) | 1 (reference) |
|  | 1-499 | 956309 | 4891 | 7922856.8 | 0.617 | 0.60 (0.58, 0.62) | 0.82 (0.79, 0.85) | 0.85 (0.81, 0.88) |
|  | 500-999 | 773347 | 4470 | 6410633.48 | 0.697 | 0.68 (0.65, 0.71) | 0.81 (0.78, 0.85) | 0.84 (0.81, 0.88) |
|  | 1000-1499 | 268990 | 1678 | 2233896.68 | 0.751 | 0.73 (0.69, 0.77) | 0.77 (0.73, 0.82) | 0.81 (0.77, 0.86) |
|  | ≥1500 | 120551 | 1020 | 999462.18 | 1.020 | 0.99 (0.93, 1.06) | 0.82 (0.76, 0.87) | 0.85 (0.80, 0.91) |
| *P* for trend |  |  |  |  |  | <0.001 | <0.001 | <0.001 |
| 1,2 | Totally sedentary | 1122450 | 28611 | 9104182.69 | 3.142 | 3.05 (2.96, 3.15) | 1.64 (1.59, 1.69) | 1.64 (1.59, 0.69) |
|  | 1-499 | 1517237 | 24341 | 12439460.87 | 1.956 | 1.90 (1.84, 1.96) | 1.40 (1.36, 1.45) | 1.43 (1.39, 1.48) |
|  | 500-999 | 1294867 | 22704 | 10612087.89 | 2.139 | 2.08 (2.02, 2.14) | 1.35 (1.31, 1.39) | 1.39 (1.34, 1.43) |
|  | 1000-1499 | 490913 | 8708 | 4029702.15 | 2.160 | 2.10 (2.03, 2.17) | 1.28 (1.23, 1.32) | 1.34 (1.30, 1.39) |
|  | ≥1500 | 242208 | 5770 | 1975517.27 | 2.920 | 2.84 (2.73, 2.95) | 1.32 (1.27, 1.37) | 1.40 (1.34, 1.45) |
| *P* for trend |  |  |  |  |  | <0.001 | <0.001 | <0.001 |
| 3,4 | Totally sedentary | 527190 | 25330 | 4206742.25 | 6.021 | 5.85 (5.68, 6.03) | 2.22 (2.16, 2.29) | 2.22 (2.16, 2.29) |
|  | 1-499 | 624978 | 21295 | 5058323.85 | 4.209 | 4.09 (3.97, 4.22) | 1.99 (1.93, 2.06) | 2.03 (1.97, 2.09) |
|  | 500-999 | 548555 | 19387 | 4436721.88 | 4.369 | 4.25 (4.12, 4.38) | 1.88 (1.82, 1.94) | 1.93 (1.87, 1.99) |
|  | 1000-1499 | 214393 | 7189 | 1738992.09 | 4.134 | 4.02 (3.87, 4.16) | 1.72 (1.66, 1.78) | 1.80 (1.74, 1.87) |
|  | ≥1500 | 112794 | 4586 | 907965.43 | 5.050 | 4.91 (4.71, 5.11) | 1.70 (1.63, 1.77) | 1.79 (1.72, 1.87) |
| *P* for trend |  |  |  |  |  | <0.001 | <0.001 | <0.001 |
| 5 | Totally sedentary | 64190 | 4432 | 505315.17 | 8.770 | 8.54 (8.20, 8.89) | 2.67 (2.56, 2.78) | 2.67 (2.57, 2.78) |
|  | 1-499 | 66613 | 3516 | 531782.47 | 6.611 | 6.43 (6.16, 6.71) | 2.38 (2.28, 2.49) | 2.42 (2.32, 2.53) |
|  | 500-999 | 58901 | 3091 | 470651.24 | 6.567 | 6.39 (6.11, 6.68) | 2.21 (2.11, 2.31) | 2.28 (2.18, 2.38) |
|  | 1000-1499 | 22960 | 1091 | 184451.05 | 5.914 | 5.75 (5.38, 6.14) | 1.99 (1.86, 2.13) | 2.09 (1.96, 2.24) |
|  | ≥1500 | 13116 | 798 | 104494.76 | 7.636 | 7.42 (6.89, 8.00) | 2.17 (2.02, 2.34) | 2.29 (2.13, 2.47) |
| *P* for trend |  |  |  |  |  | <0.001 | <0.001 | <0.001 |

MET, metabolic equivalent of tasks

Multivariate model 1 was adjusted for age and sex.

Multivariate model 2 was adjusted for age, sex, smoking, alcohol consumption, and body mass index

**Additional Figure 1.** The multivariable-adjusted risk for (a) all-cause mortality and (b) incident cardiovascular disease by physical activity amount according to the presence of metabolic syndrome after adjustment for age, sex, smoking status, alcohol consumption, and body mass index.

HR, hazard ratio; CVD, cardiovascular disease; MS, metabolic syndrome; MET, metabolic equivalent


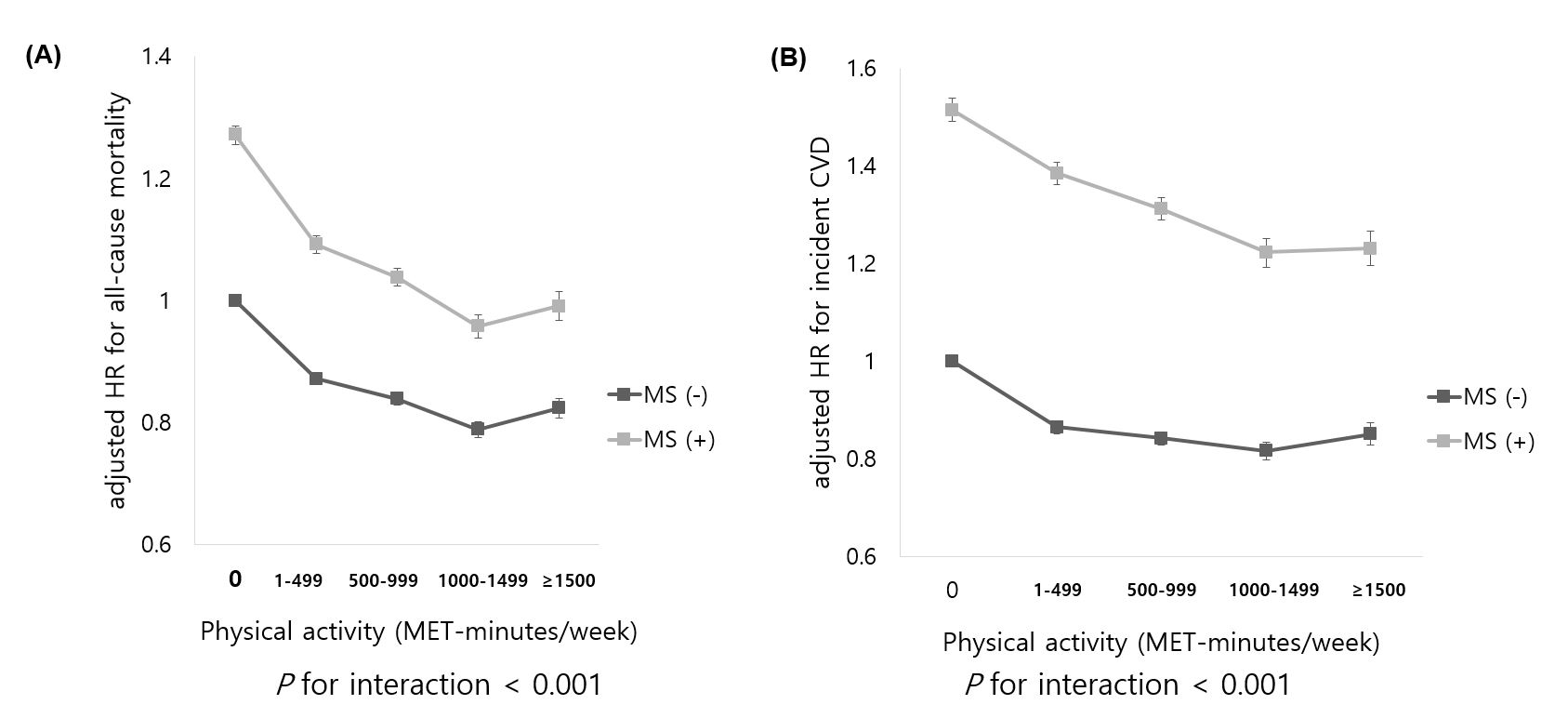

Supplement: Supplementary file 1 — Additional file 1: Table S1. Questionnaires on the habits of leisure-time physical activity evaluated during routine health check-ups by National Health Insurance Service. Table S2. Baseline characteristics according to the presence of metabolic syndrome. Table S3. The effect of the number of metabolic syndrome components and physical activity amount on all-cause mortality. Table S4. The effect of the number of metabolic syndrome components and PA amount on cardiovascular disease. Figure S1. The multivariable-adjusted risk for (a) all-cause mortality and (b) incident cardiovascular disease by physical activity amount according to the presence of metabolic syndrome after adjustment for age, sex, smoking status, alcohol consumption, and body mass index. [file 13098_2023_1227_MOESM1_ESM.docx]
